# TCM splints versus internal fixation for distal radius fractures: A systematic review and meta-analysis of randomized controlled trials

**DOI:** 10.1097/MD.0000000000043366

**Published:** 2025-07-18

**Authors:** Wantao Xiong, Xin Cui, Junchen Li, Chenqi Guo, Yizhuo Yan, Yongyao Li

**Affiliations:** a Wangjing Hospital of China Academy of Chinese Medical Sciences, Beijing, China; b Institute of Basic Research in Clinical Medicine, China Academy of Chinese Medical Sciences, Beijing, China; c Tianjin University of Traditional Chinese Medicine, Tianjin, China; d Tianjin Medical College, Tianjin, China.

**Keywords:** distal radius fractures, internal fixation, meta-analysis, splints, systematic review

## Abstract

**Background:**

The optimal treatment of distal radius fractures (DRFs) remains unclear. This study aimed to compare the clinical outcomes of traditional Chinese medicine (TCM) splints and internal fixation in the treatment of DRFs.

**Methods:**

A comprehensive search was conducted for randomized controlled trials comparing TCM splints with internal fixation for DRFs using databases such as PubMed, Web of Science, EMBASE, Cochrane Library, SinoMed, the Chinese National Knowledge Infrastructure Database, Wanfang Database, and VIP Database. Following literature screening, data extraction, and evaluation of the risk of bias, data analysis was performed using RevMan 5.3 and Stata 14.2 software. The quality of evidence for each outcome was assessed using the Grading of Recommendations Assessment, Development, and Evaluation system.

**Results:**

Eighteen studies comprising 1682 patients with DRFs were included in this analysis. No significant differences were observed in clinical effective rate, volar tilt, radial inclination, radial height, visual analog scale score, or complication rate between the treatment of TCM splints and internal fixation for DRFs. In addition, for clinical effective rate, subgroup analysis based on different evaluation criteria showed no significant difference between the groups. TCM splints demonstrated advantages in fracture healing time.

**Conclusion:**

TCM splints and internal fixation methods are similar in most clinical outcomes in the treatment of DRFs. However, TCM splints exhibited superior performance in terms of fracture healing time, making them a preferable option. Nonetheless, TCM splints are not a replacement for internal fixation, and internal fixation should still be performed when there are surgical indications. When considering internal fixation surgery, it is critical to thoroughly evaluate the risks and benefits. The optimal treatment plan should be tailored based on the patient’s specific needs, individual circumstances, and available clinical expertise.

## 1. Introduction

Distal radius fractures (DRFs) occur on the outer edge near the pronator muscle’s proximal terminus, situated at the interface of the dense and cancellous bones of the distal radius, which is prone to damage from external forces. DRFs account for approximately 17.5% of all fractures, mostly in middle and older age groups.^[1,2]^ As global populations age, the prevalence of DRFs among the elderly continues to escalate, positioning these injuries among the top 3 fractures in this demographic. Moreover, due to increased industrial activity worldwide, the rise in high-energy traumas, such as vehicular accidents and falls from significant heights, has led to a gradual increase in prevalence among younger individuals.^[3,4]^ Treatment modalities for DRFs range from conservative approaches to surgical interventions, determined by the fracture’s characteristics, severity, patient’s age, and joint mobility.^[5]^ Traditional Chinese medicine (TCM) splints external fixation is a classical and reliable therapeutic method for conservative treatment, which has a number of advantages such as noninvasiveness, simplicity, affordability, and the elimination of hospital stay, often accelerating recovery and minimizing complications associated with DRFs.^[6–8]^ The representative diagram of TCM splints intervention for DRFs is provided in Figure S1, Supplemental Digital Content, https://links.lww.com/MD/P482. Conversely, surgical treatments provide high precise reductions, crucial for restoring wrist anatomy and facilitating early functional exercise, thereby improving wrist function.^[9,10]^ However, the debate between the efficacy of TCM splints external fixation and surgical internal fixation complicates clinical decision-making.^[11–13]^ This study aims to systematically review and meta-analyze randomized controlled trials (RCTs) comparing TCM splints with surgical internal fixation in the treatment of DRFs, thereby guiding clinicians toward the most effective treatment strategies.

## 2. Materials and methods

### 2.1. Registration

This meta-analysis was conducted according to the Preferred Reporting Items for Systematic Reviews and Meta-Analyses (PRISMA).^[14]^ The research protocol was registered in PROSPERO on April 17, 2024, with the registration website https://www.crd.york.ac.uk/prospero/#recordDetails and registration number CRD42024532741.

### 2.2. Search strategy

We searched 8 electronic databases including PubMed, Web of Science, EMBASE, Cochrane Library, SinoMed, the Chinese National Knowledge Infrastructure Database, Wanfang Database, and VIP Database. All databases were searched from inception to March 31, 2024, with no restrictions on publication date or language. The key terms used in these searches were radius fractures, Colles fracture, Smith fracture, Barton fracture, wrist injuries, splints, static splints, dynamic splints, internal fixation, and RCTs in both Chinese and English. The search strategy for PubMed is described in Appendix 1, Supplemental Digital Content, https://links.lww.com/MD/P481. In addition, we manually searched the reference lists of the included studies or any other relevant publications to identify other eligible studies.

### 2.3. Inclusion and exclusion criteria

The criteria for inclusion of the studies included: (1) the type of studies included was RCTs. (2) DRF patients were required to have a clear imaging diagnosis, regardless of gender, age, race, nationality, primary disease, or clinical stage. (3) The studies directly compared TCM splints and internal fixation management. The intervention of the experimental group was external fixation with TCM splints. The intervention for the control group was internal fixation. (4) Main outcome was clinical effective rate, and secondary outcomes were radiographic parameters after treatment including volar tilt, radial inclination and radial height, fracture healing time, complications, and visual analog scale (VAS) score. The clinical effective rate was evaluated using the following 3 methods: wrist Cooney score,^[15]^ Gartland–Werley score,^[16]^ and Dinest score.^[17]^ Cooney score included 4 parts, pain symptoms, functional status, grip strength, and range of motion. The evaluation criteria were as follows: excellent, 90 to 100 points; good, 80 to 89 points; fair, 65 to 79 points; poor, 0 to 64 points. Gartland–Werley score, which is based on a demerit point system, are awarded for pain, deformity, range of motion, and complication: excellent, 0 to 2 points; good, 3 to 8 points; fair, 9 to 20 points; poor, more than 20 points. Dinest score, which includes reduction in palm or dorsiflexion, grip strength, movement, pain, and function, is graded as excellent, good, fair, or poor. We define the clinical effective rate = (patients number of “excellent” function + number of “good” function patients+“fair” function)/total overall patients number × 100%. Exclusion criteria were: (1) studies without definitive diagnostic criteria or specific randomized methods. (2) Studies that were not available in full text, were repeatedly republished, contained unpublished trial data, or were conference papers.

### 2.4. Literature screening and data extraction

The first step was to import all the identified catalogue information into the literature management software and then remove any duplicate papers. After that, a preliminary selection was made by reading the title and abstract. Finally, the literature that did not meet the inclusion criteria was eliminated by reading the full text to determine which literature should be included in the analysis. Reasons for unqualified or excluded documents were recorded. Two reviewers (Xin Cui, Junchen Li) independently extracted data from the included literature. In case of any discrepancies between the 2 reviewers, a third reviewer (Chenqi Guo) was consulted. The following data were extracted: title, author, year of publication, sample size, intervention, follow-up, clinical effective rate, radiographic parameters, fracture healing time, complications, and VAS score, among others.

### 2.5. Risk of bias assessment

The methodological quality of the included studies was assessed according to the “risk of bias table” of the Cochrane Collaboration.^[18]^ Two reviewers (Xin Cui, Chenqi Guo) independently evaluated the risk of bias and quality of evidence for all outcomes, while a third reviewer (Junchen Li) resolved any possible discrepancies. The evaluation considered the following 7 aspects: random sequence generation, allocation concealment, blinding of investigators and/or subjects, blinding of study results evaluators, integrity of results data, selective reporting, and other biases.

### 2.6. Grading the quality of evidence

In order to evaluate the quality of evidence for each outcome of the meta-analysis, we used the Grading of Recommendations Assessment, Development, and Evaluation method,^[19]^ which recommended that the quality of evidence could be classified into 4 levels: high (++++), moderate (+++), low (++), and very low (+). We assessed it on this website: https://gradepro.org/.

### 2.7. Statistical analysis

RevMan 5.4 and Stata 14.2 software were utilized for conducting the meta-analysis. For dichotomous variables, the risk ratio (RR) along with 95% confidence interval (CI) were calculated. For continuous variables, mean difference and 95% CI were used. To assess heterogeneity among the included studies, the Cochran Q test and I² statistic were employed. In instances where statistical homogeneity was observed between studies (*P* > .10 and I² < 50%), a fixed-effect model was applied for the meta-analysis. Otherwise, the random effects model was adopted. Furthermore, STATA 14.2 software was used for sensitivity analysis to explore the stability of the outcomes. A funnel plot for evaluating publication bias was employed if there were 10 or more included studies.

## 3. Results

**Figure 1.**
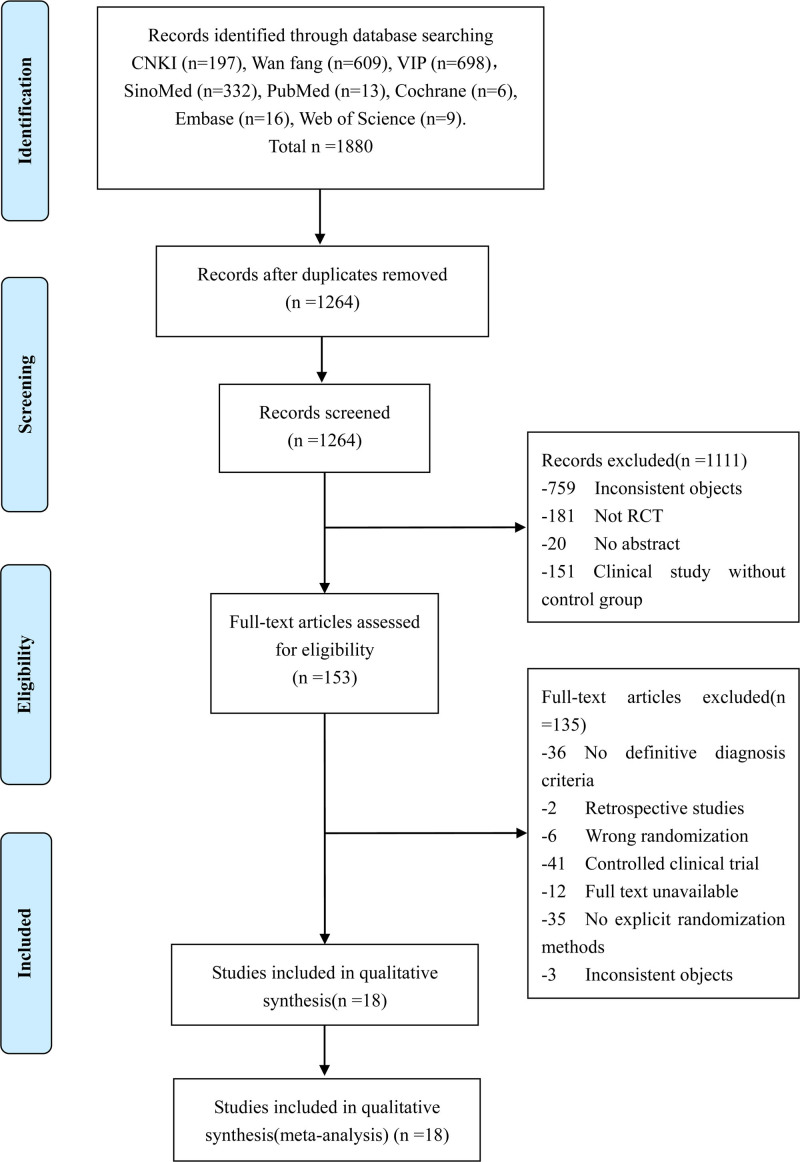
Flow diagram of study selection.

The literature screening process and results are shown in Figure 1. A total of 1880 literatures were retrieved, and 18 qualified literatures were selected for inclusion in the meta-analysis after preliminary screening and full-text reading.

### 3.1. Characteristics of the included studies

A total of 18 studies^[8,11–13,20–33]^ were included in this analysis, involving a total of 1682 patients, of whom 844 were in the experimental group and 838 were in the control group. All included studies were conducted in China. The clinical effective rate mainly has the following 3 evaluation methods: wrist Cooney score,^[17]^ Gartland–Werley score,^[18]^ and Dinest score.^[19]^ In 17 studies,^[8,11–13,20–25,27–33]^ clinical effective rate were reported, out of which 5 studies^[8,27,30–32]^ were based on wrist Cooney scores, 6 studies^[11–13,22,23,29]^ were based on Gartland–Werley score, and 3 studies^[20,21,24]^ were based on Dinest score. The remaining 3 studies^[25,28,33]^ did not meet the above 3 criteria, and including them would result in significant statistical heterogeneity. Therefore, to ensure the rigor of this analysis, the clinical effective rate results of these 3 studies were not included. Radiographic parameters were reported in 7 studies,^[8,12,21,22,26,29,31]^ all of which reported volar tilt and radial inclination, but only 3 of them^[22,26,29]^ reported radial height. Fracture healing time was reported in 8 studies,^[8,12,23,26,28–31]^ complication rate was reported in 10 studies,^[12,13,20,22,23,25,26,28,30,32]^ and VAS score was reported in 5 studies.^[13,20,26,27,29]^ The characteristics of the included studies are shown in Table 1.

**Table 1:** Study characteristics.

References	Sample size E/C (M/F)	Age (years) E/C	Intervention E/C	Follow-up	Main outcomes
Zhu^[20]^	35/38 (26/47)	44.6 ± 4.0	Splints/plate		①⑥⑦
Gao^[21]^	E: 31 (14/17) C: 30 (14/16)	E: 65.85 ± 3.95 C: 65.48 ± 3.79	Splints/plate	3–18 mo	①②③
Yan^[11]^	E: 108 (60/48) C: 108 (61/47)	E: 54.2 ± 11.3 C: 54.8 ± 10.6	Splints/plate	3 mo	①
Li^[22]^	E: 38 (22/16) C: 38 (21/17)	E: 63.60 ± 4.15 C: 63.54 ± 4.26	Splints/plate		①②③④⑥
Lao^[23]^	E: 126 (26/100) C: 118 (24/94)	E: 71.2 ± 12.2 C: 70.7 ± 13.9	Splints/plate	1.5–12 mo	①⑤⑥
Liu^[8]^	E: 30 (13/17) C: 30 (12/18)	E: 46.82 ± 4.57 C: 47.18 ± 4.63	Splints/plate		①②③⑤
Cai^[24]^	E: 70 (30/40) C: 70 (27/43)	E: 58.2 ± 3.3 C: 59.2 ± 4.1	Splints/plate		①
Yan^[25]^	E: 23 (15/8) C: 23 (13/10)	E: 62.18 ± 1.06 C: 62.14 ± 1.20	Splints/Kirschner wire		①⑥
Liu^[26]^	E: 30 (19/11) C: 30 (18/12)	E: 65.41 ± 4.70 C: 65.29 ± 4.65	Splints/plate	3–6 mo	②③④⑤⑥⑦
Chen^[27]^	E: 35 (14/21) C: 35 (15/20)	E: 68.35 ± 3.12 C: 69.13 ± 3.76	Splints/plate		①⑦
Ye^[28]^	E: 40 (19/21) C: 40 (22/18)	E: 63.09 ± 7.19 C: 62.95 ± 6.43	Splints/plate	2–3 mo	①⑤⑥
Liu^[29]^	E: 50 (28/22) C: 50 (29/21)	E: 65.29 ± 2.53 C: 64.86 ± 2.72	Splints/plate		①②③④⑤⑦
Guo^[30]^	E: 43 (24/19) C: 42 (22/20)	E: 55.63 ± 5.05 C: 55.65 ± 4.80	Splints/plate	6 mo	①⑤⑥
Wang^[31]^	49/49 (45/53)	67.1 ± 2.8	Splints/plate	1.5–3 mo	①②③⑤
Liang^[32]^	E: 30 (18/12) C: 30 (16/14)	E: 50.2 ± 2.1 C: 50.2 ± 2.2	Splints/plate		①⑥
Xu^[33]^	E: 41 (23/18) C: 40 (25/15)	E: 71.2 ± 2.3 C: 70.8 ± 2.1	Splints/Kirschner wire	6 mo	①
Wu^[12]^	E: 41 (22/19) C: 41 (21/20)	E: 69.5 ± 1.4 C: 69.6 ± 1.3	Splints/Kirschner wire	3 mo	①②③⑤⑥
Sun^[13]^	E: 24 (10/14) C: 26 (9/17)	E: 74.56 ± 4.14 C: 75.17 ± 5.26	Splints/plate	3 mo	①⑥⑦

①: Clinical effective rate; ②: volar tilt; ③: radial inclination; ④: radial height; ⑤: fracture healing time; ⑥: complications; ⑦: VAS. C = control group, E = experimental group.

### 3.2. Results of the risk of bias

**Figure 2.**
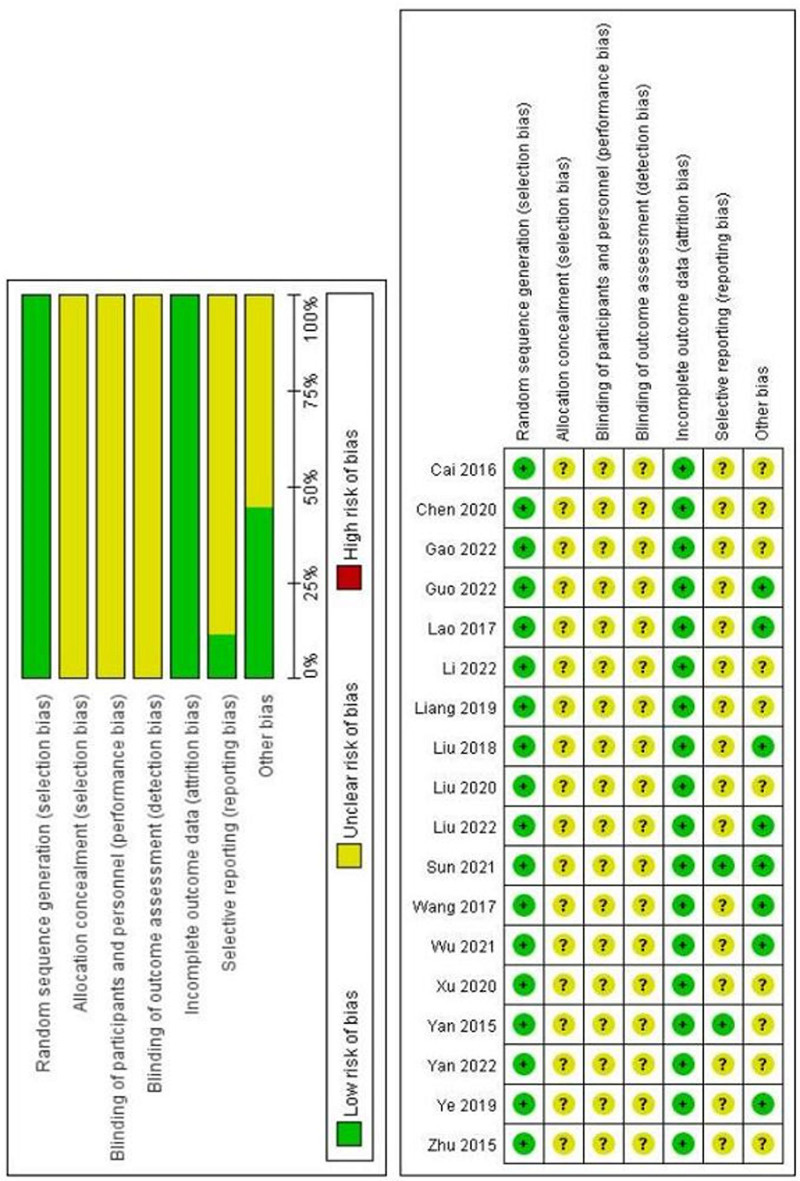
Results of the risk of bias evaluation.

All the studies included used various methods to generate random sequences. Sixteen studies^[8,11–13,20–24,26–31,33]^ used the random number table method, 1 study^[25]^ used the 2-color sphere method, and 1 study^[32]^ used the random comprehensive sequential method. None of the included studies explicitly mentioned allocation concealment and blinding. No outcome data was missing in any of the studies. Two studies^[11,13]^ did not report selectivity, and it is unclear about selectivity reporting for the remaining 16 studies.^[8,12,20–33]^ There were 8 studies^[12,13,23,26,28–31]^ with no other risk of bias and the remaining 8 studies^[8,11,20–22,27,32,33]^ with other possible risks. The results of the bias risk evaluation are presented in Figure 2.

### 3.3. Meta‑analysis of clinical results

#### 3.3.1. Clinical effective rate

**Figure 3.**
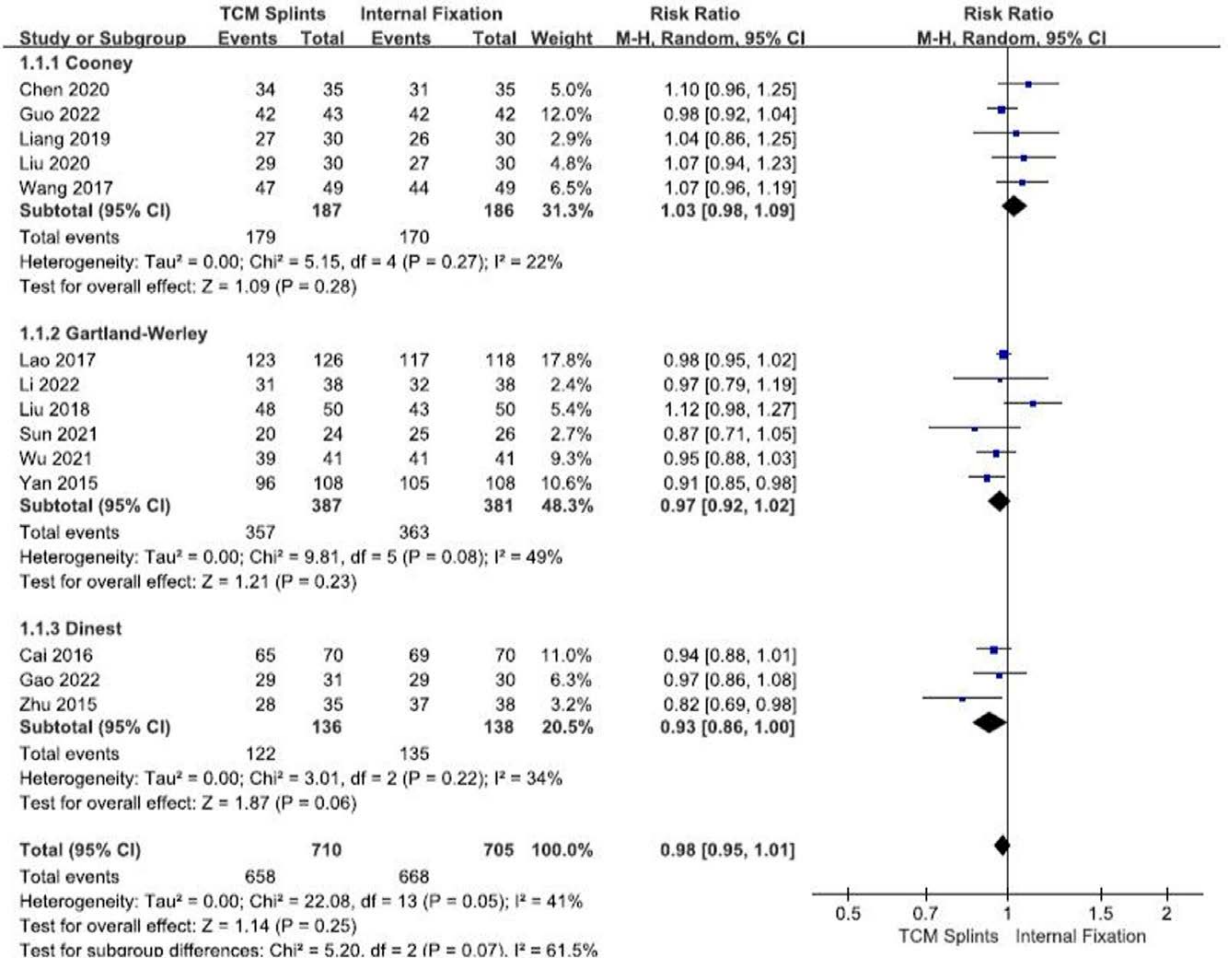
Forest plot for clinical effective rate.

A total of 17 RCTs^[8,11–13,20–25,27–33]^ were included, involving 1415 patients, including 710 in the experimental group and 705 in the control group. There was some heterogeneity among the studies (I^2^ = 41%, *P* = .05). The results of random effects model meta-analysis showed that: [RR = 0.98, 95% CI (0.95, 1.01), *P* = .25] (Fig. 3), suggesting that there was no significant difference in the clinical effective rate of TCM splints and internal fixation in the treatment of DRFs. Subgroup analysis was conducted according to different evaluation criteria, and the results showed that the clinical effective rate of the 2 interventions in each subgroup was not statistically significant (Fig. 3).

#### 3.3.2. Radial inclination

**Figure 4.**
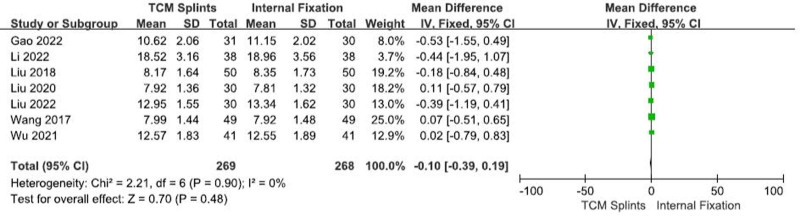
Forest plot for radial inclination.

A total of 7 RCTs^[8,12,21,22,26,29,31]^ were included, involving 537 patients, including 269 in the experimental group and 268 in the control group. There was good homogeneity among the studies (I^2^ = 0%, *P* = .90). Meta-analysis using the fixed-effect model showed that: [RR = 0.10, 95% CI (-0.39, 0.19), *P* = .48] (Fig. 4), suggesting that there was no significant difference in radial inclination between the 2 groups after TCM splints and internal fixation treatment of DRFs.

#### 3.3.3. Volar tilt

**Figure 5.**
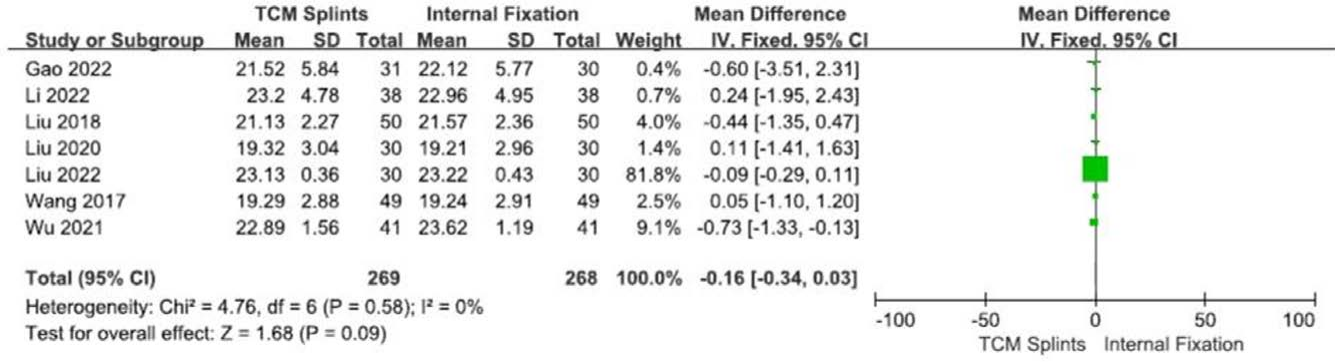
Forest plot for volar tilt.

Seven RCTs^[8,12,21,22,26,29,31]^ with a total of 537 patients were analyzed, with 269 in the experimental group and 268 in the control group. The studies presented good homogeneity (I^2^ = 0%, *P* = .58). Meta-analysis was conducted using the fixed-effect model showed that: [RR = -0.16, 95% CI (-0.34, 0.03), *P* = .09] (Fig. 5), suggesting that there was no significant difference in the volar tilt between the 2 groups after the treatment of DRFs with TCM splints and internal fixation.

#### 3.3.4. Radial height

**Figure 6.**
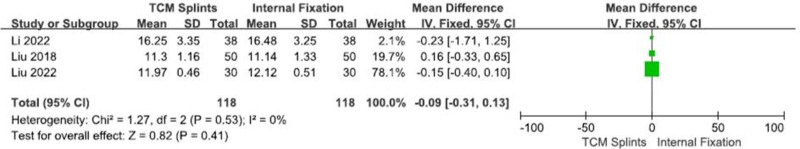
Forest plot for radius height.

A total of 3 RCTs^[22,26,29]^ were analyzed, which included a total of 236 patients, with 118 in the experimental group and 118 in the control group. The studies were found to be homogeneous (I^2^ = 0%, *P* = .53). The meta-analysis results, using the fixed-effect model, showed that there was no significant difference in radius height between the 2 groups after the treatment of DRFs with TCM splints and internal fixation [RR = -0.09, 95% CI (-0.31, 0.13), *P* = .41] (Fig. 6).

#### 3.3.5. Fracture healing time

**Figure 7.**
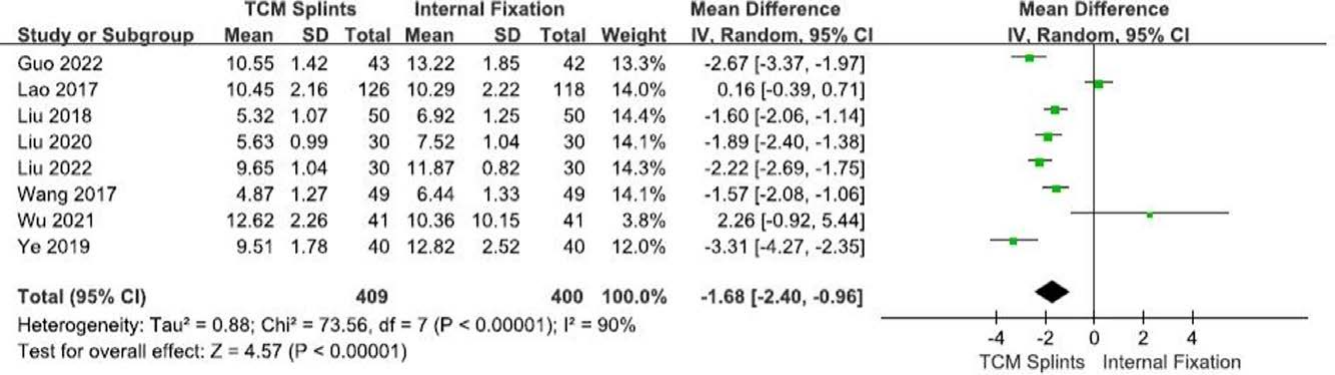
Forest plot for fracture healing time.

A total of 8 RCTs^[8,12,23,26,28–31]^ were included, which involved 809 patients, including 409 in the experimental group and 400 in the control group. Heterogeneity among studies was high (I^2^ = 90%, *P* < .00001). The results of meta-analysis using random effects model showed that: [RR = -1.68, 95% CI (-2.40, -0.96), *P* < .00001] (Fig. 7), suggesting that TCM splints can significantly shorten the fractures healing time compared with internal fixation.

#### 3.3.6. Complications

**Figure 8.**
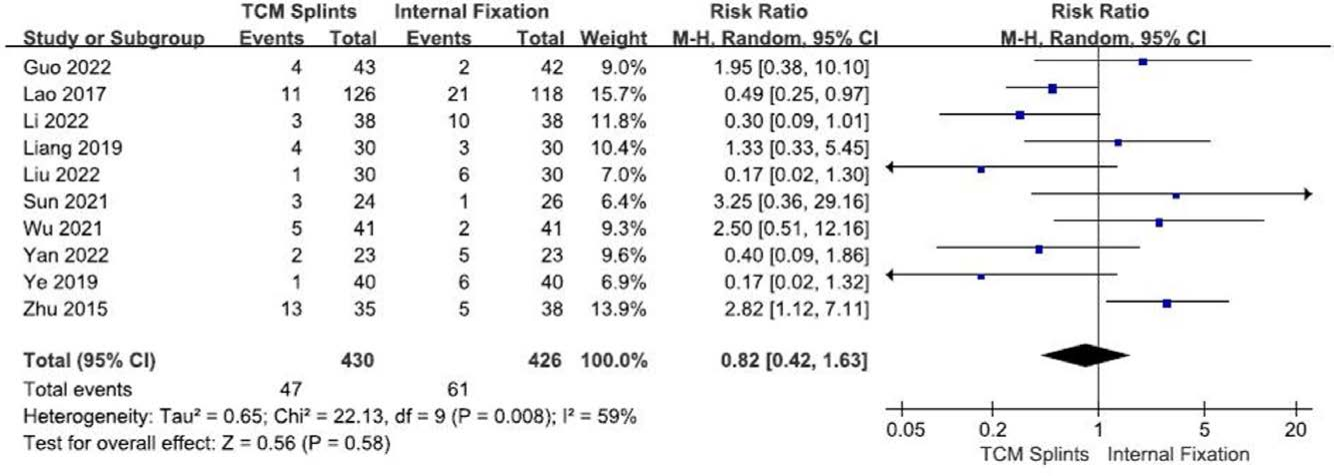
Forest plot for complication rate.

A total of 10 RCTs^[12,13,20,22,23,25,26,28,30,32]^ were included, involving 856 patients, including 430 in the experimental group and 426 in the control group. There was certain heterogeneity among studies (I^2^ = 59%, *P* = .008). The results of meta-analysis using random effects model showed: [RR = 0.82, 95% CI (0.42, 1.63), *P* = .58] (Fig. 8), suggesting that there was no significant difference in the incidence of complications between the 2 groups after TCM splints and internal fixation were used to treat DRFs.

#### 3.3.7. VAS

**Figure 9.**
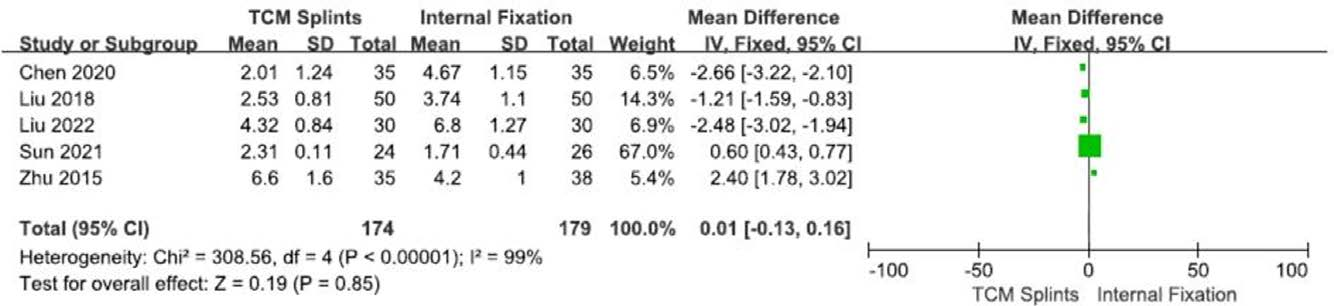
Forest plot for VAS score. VAS = visual analog scale.

A total of 5 RCTs^[13,20,26,27,29]^ were included, involving 353 patients, including 174 in the experimental group and 179 in the control group. The heterogeneity between studies is very high (I^2^ = 99%, *P* < .00001). The results of meta-analysis using random effects model showed: [RR = 0.01, 95% CI (-0.13, 0.16), *P* = .85] (Fig. 9), indicating that there was no significant difference in VAS score between the 2 groups after the treatment of DRFs with TCM splints and internal fixation.

### 3.4. Sensitivity analysis

**Figure 10.**
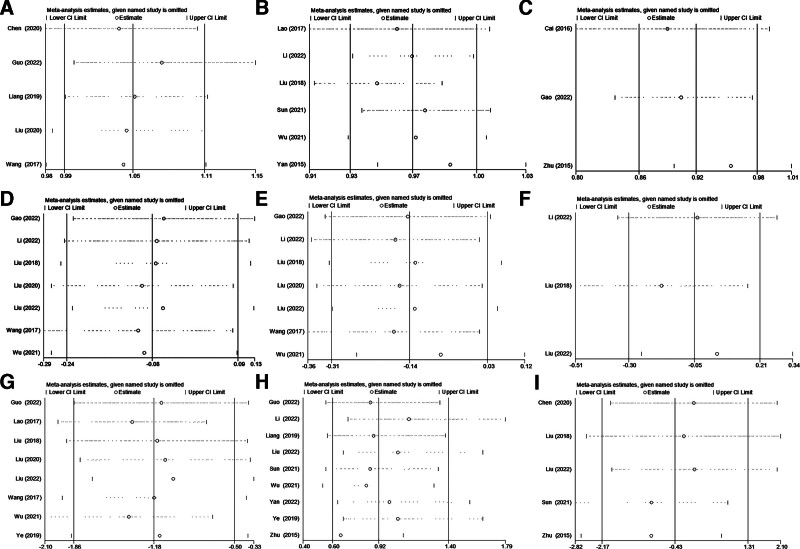
The results of sensitivity analysis. (A) Cooney score, (B) Gartland–Werley score, (C) Dinest score, (D) volar tilt, (E) radial inclination, (F) radial height, (G) fracture healing time, (H) complications, and (I) VAS score. VAS = visual analog scale.

During the analysis of the outcome indicators, each individual study was excluded one by one. It was observed that there were no significant influences on any outcome, which indicates that the sensitivity was low and the results were robust. The details can be found in Figure 10.

### 3.5. Assessment of publication bias

**Figure 11.**
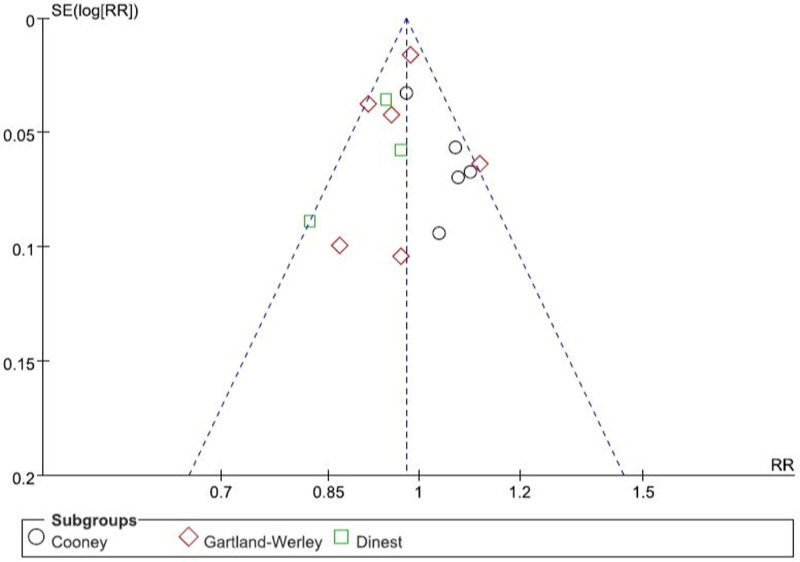
Funnel plot of clinical effective rate.

Publication bias was analyzed by clinical effective rate which included most studies, and a funnel plot was drawn. As shown in Figure 11, the symmetry of the plot indicated that publication bias was not obvious.

### 3.6. Level of evidence

The Grading of Recommendations Assessment, Development, and Evaluation level of evidence is moderate for radial inclination, volar tilt and clinical effective rate of Gartland–Werley score. The evidence of clinical effective rate, clinical effective rate of Cooney and Dinest score, radial height, and fracture healing time was identified as low level. The level of evidence of complications and VAS was assessed as very low. Details can be found in Table S1, Supplemental Digital Content, https://links.lww.com/MD/P483.

## 4. Discussion

This study comprehensively evaluated the efficacy and safety of TCM splints versus internal fixation in treating DRFs. Parameters after treatment such as clinical effective rate, radiographic parameters (radial inclination, volar tilt, and radial height), fracture healing time, and VAS score were employed to assess the effectiveness of both treatments in DRF patients. The incidence of complications provided a measure of safety for the 2 interventions. The results revealed no significant differences in clinical efficacy, imaging outcomes, or VAS score between TCM splints and internal fixation methods. Moreover, long-term follow-up showed that, compared to internal fixation surgery, TCM splints external fixation notably reduced fracture healing time but there was no significant difference in complication rate.

The treatment of DRFs through the use of splints external fixation represents the essence of traditional Chinese orthopedics, characterized by its long-standing history, reliable efficacy, and cost-effectiveness. TCM splints generally choose wood, or bamboo as a material, often divided into palmar, dorsal, ulnar, radial splints 4, dorsal plate distal to the 2nd, 3rd, 4th metacarpal bone base, palmar plate distal to the wrist joint, radial plate distal to the first metacarpal base, ulnar plate distal to the fifth metacarpal base, bundled requires the ability to lift the tie in the splint up and down to move 1cm, forming an elastic fixed.^[34,35]^ In contrast, internal fixation surgeries focus on anatomical reduction and robust stabilization, offering enhanced stability that enables quicker healing and earlier commencement of functional rehabilitation.^[36]^

The results of this study indicated that there were no significant differences in clinical effective rate, radiographic parameters, VAS score, or complication rate between TCM splints and internal fixation surgery, and TCM splints demonstrated advantages in fracture healing time. The reasons may be as follows: although TCM splints external fixation therapy cannot make the fracture end achieve perfect alignment, it often enables functional reduction with manipulation and imaging cooperation. TCM splints combined with functional exercises maintain stability at the fracture site, and permit sufficient limb movement which conforms to the concept of micromobility, enhancing callus development and facilitating quicker fracture healing.^[35,37]^ On the other hand, internal fixation therapy can completely correct the displacement of the fracture to achieve anatomical repositioning, internal fixation surgery is an invasive procedure, during the operation to the affected limb of the soft tissue and blood vessels have a certain degree of damage. Part of the blood transport of the bone is accomplished by the soft tissues, and the peeling of the soft tissue and periosteum after the surgical incision will inevitably damage the blood transport, which will have a certain impact on the healing of the fracture.^[38,39]^ The study also revealed no significant difference between the 2 groups in terms of complications, and the possible reasons for this are as follows: The use of TCM splints requires immobilization for at least 4 to 6 weeks, during which extensive physical activity of wrist joints is restricted. The tightness of TCM splints during fixation is not adjusted on time, if it is too tight, the prolonged excessive compression will lead to local tissue hypoxia and blood flow obstruction. But if it is too loose, it will lose the fixation effect and make the fracture end lose stability, which may lead to delayed healing of the fracture.^[40,41]^ Additionally, material limitations in weight, breathability waterproofness fit elasticity affect recovery when using TCM splints.^[34]^ The process of internal fixation surgery causes damage to the soft tissues and blood vessels of the affected limb as well as a risk for intraoperative infection. In addition, postoperative adhesions between soft tissues or between soft tissues and bones can be caused, affecting the recovery of joint function.^[42]^

According to the meta-analysis of this study, there were no significant differences in clinical effective rate, radiographic parameters, VAS score, or complication rate between TCM splints and internal fixation surgery. Meanwhile, TCM splints demonstrated advantages in fracture healing time. Meanwhile, there recently has been an increasing amount of research on TCM splints with continuous improvements and innovations focusing on personalization and lightweight designs.^[34]^ Ongoing optimization of splints in terms of structure, material, and performance has led to the invention of innovative designs, such as 3D-printed splints and smart airbag pressure pads. These innovations have improved comfort and breathability during clinical use, while also enhancing the stability of fracture ends, which have significantly improved the clinical outcomes of traditional splints.^[43,44]^ While anatomic reduction via internal fixation may be more suitable for open distal radius fractures or those requiring high functional precision of the wrist, TCM splints external fixation offers a viable alternative for elderly patients with osteoporosis or those with multiple underlying diseases where surgical risks are a concern. TCM splints can reduce economic costs and potentially decrease the inconvenience of extended recovery periods through combined functional exercise.

This study presents several limitations. Firstly, surgical treatments such as TCM splints and internal fixation could not be blinded to subjects and investigators. While all included studies detailed specific randomization methods, none of the studies mentioned allocation concealment, potentially introducing selection bias. Secondly, the study’s sample size is relatively small, and since all included studies were conducted within China, there is a risk of bias of regional and ethnic differences potentially impacting the authenticity and accuracy of research results. Thirdly, there was considerable heterogeneity in the results regarding fracture healing time, complication rate, and VAS score. However, sensitivity analysis did not alter the outcomes of the individual studies. This heterogeneity likely stems from clinical variability, including fracture types, patient individual differences, and the clinical expertise of the investigators, as well as differences in the operational techniques and materials used for splint fixation and surgery. Fourthly, certain outcomes, such as radial height and VAS, were included in the analysis with too few references, diminishing the reliability of the study results.

## 5. Conclusions

In the comparison of fracture treatment using TCM splints and internal fixation, no significant differences were observed in most clinical outcomes. However, this study found that TCM splints exhibited superior performance in terms of fracture healing time. For DRFs, TCM splints may be preferable. Nonetheless, TCM splints are not a replacement for internal fixation, and internal fixation should still be performed when there are surgical indications. When considering internal fixation surgery, a thorough evaluation of the risks and benefits is critical. The optimal treatment plan should be tailored based on the patient’s specific needs, individual circumstances, and clinical expertise. However, the results of this study are subject to bias due to the quality of the original research, necessitating further high-quality, multicenter, large-sample prospective RCTs to validate and support the conclusions.

## Author contributions

**Conceptualization:** Wantao Xiong.

**Data curation:** Xin Cui, Junchen Li.

**Formal analysis:** Xin Cui, Chenqi Guo, Yizhuo Yan.

**Project administration:** Yongyao Li.

**Writing – original draft:** Wantao Xiong.

**Writing – review & editing:** Wantao Xiong.

## Supplementary Material


