# Supplementary figures and images for: TCM splints versus internal fixation for distal radius fractures: A systematic review and meta-analysis of randomized controlled trials
Source: Medicine (Baltimore). 2025 Jul 18;104(29):e43366. doi: 10.1097/MD.0000000000043366 (PMC12282772; doi:10.1097/MD.0000000000043366)

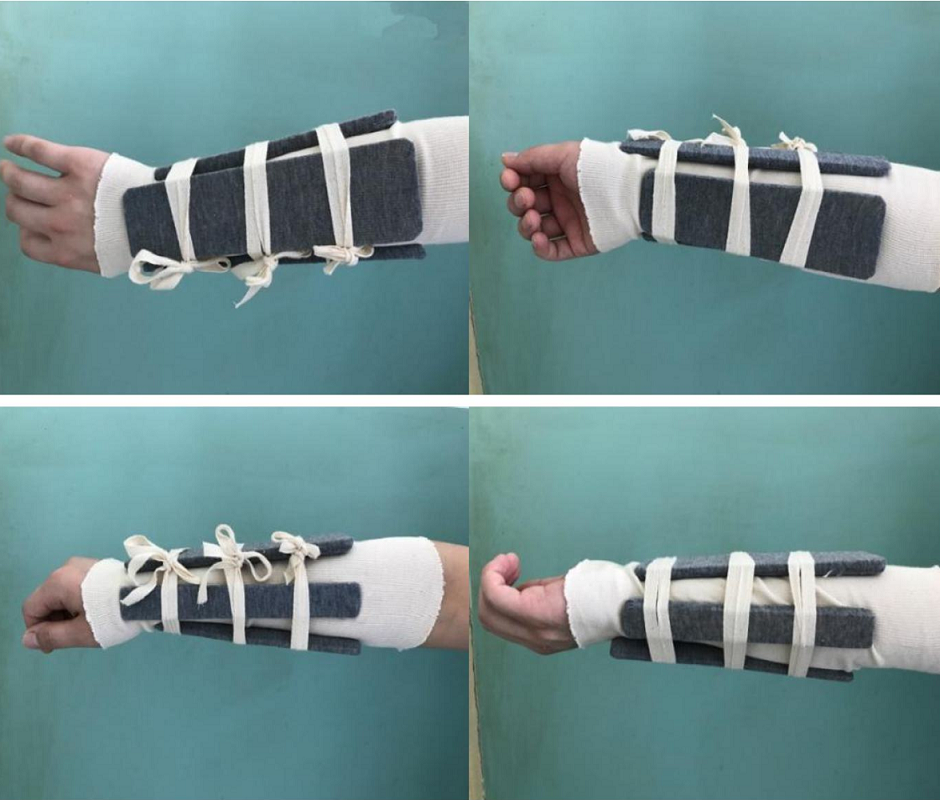

Supplement: Supplementary file 1 [file medi-104-e43366-s001.tiff]
