# Supplementary material for: TCM splints versus internal fixation for distal radius fractures: A systematic review and meta-analysis of randomized controlled trials
Source: Medicine (Baltimore). 2025 Jul 18;104(29):e43366. doi: 10.1097/MD.0000000000043366 (PMC12282772; doi:10.1097/MD.0000000000043366)
Supplement: Supplementary file 2 [file medi-104-e43366-s002.doc]

**Appendix 1**

The search strategy for PubMed

#1 (((radius fractures[MeSH Terms]) AND (distal[Title/Abstract])) OR (colles' fracture[MeSH Terms])) OR (wrist injuries[MeSH Terms])

#2 (((radius[Title/Abstract]) OR (radial[Title/Abstract])) AND (distal[Title/Abstract])) AND (fractur*[Title/Abstract])

#3 ((((colles[Title/Abstract]) OR (smith[Title/Abstract])) OR (barton[Title/Abstract])) OR (wrist[Title/Abstract])) AND (fractur*[Title/Abstract])

#4 #1 OR #2 OR #3

(((((radius fractures[MeSH Terms]) AND (distal[Title/Abstract])) OR (colles' fracture[MeSH Terms])) OR (wrist injuries[MeSH Terms])) OR ((((radius[Title/Abstract]) OR (radial[Title/Abstract])) AND (distal[Title/Abstract])) AND (fractur*[Title/Abstract]))) OR (((((colles[Title/Abstract]) OR (smith[Title/Abstract])) OR (barton[Title/Abstract])) OR (wrist[Title/Abstract])) AND (fractur*[Title/Abstract]))

#5 (((((((((((((((((splints[MeSH Terms]) OR (Splint[Title/Abstract])) OR (Static Splints[Title/Abstract])) OR (Splint, Static[Title/Abstract])) OR (Splints, Static[Title/Abstract])) OR (Static Orthoses[Title/Abstract])) OR (Static Orthose[Title/Abstract])) OR (Static Splinting[Title/Abstract])) OR (Splinting, Static[Title/Abstract])) OR (Static Splint[Title/Abstract])) OR (Dynamic Splints[Title/Abstract])) OR (Splint, Dynamic[Title/Abstract])) OR (Splints, Dynamic[Title/Abstract])) OR (Dynamic Orthoses[Title/Abstract])) OR (Dynamic Splint[Title/Abstract])) OR (Dynamic Splinting[Title/Abstract])) OR (Dynamic Splintings[Title/Abstract])) OR (Splinting, Dynamic[Title/Abstract])

#6 (Fracture Fixation, Internal[MeSH Terms]) OR (‘Fixation, Internal Fracture’[Title/Abstract] OR ‘Fixations, Internal Fracture’[Title/Abstract] OR ‘Fracture Fixations, Internal’[Title/Abstract] OR ‘Internal Fracture Fixation’[Title/Abstract] OR ‘Internal Fracture Fixations’[Title/Abstract] OR ‘Osteosynthesis, Fracture’[Title/Abstract] OR ‘Fracture Osteosyntheses’[Title/Abstract] OR ‘Fracture Osteosynthesis’[Title/Abstract] OR ‘Osteosyntheses, Fracture’[Title/Abstract])

#7 #4 AND #5 AND #6

#7 ((((((randomized controlled trial[MeSH Terms]) OR (randomized controlled trial[Publication Type])) OR (randomized controlled trial[All Fields])) OR (RCT[Title/Abstract])) OR (random allocation[Title/Abstract])) OR (Controlled Clinical Trial[Publication Type])) OR (Controlled Clinical Trial[All Fields])

#8 #4 AND #5 AND #6
