# Supplementary material for: TCM splints versus internal fixation for distal radius fractures: A systematic review and meta-analysis of randomized controlled trials
Source: Medicine (Baltimore). 2025 Jul 18;104(29):e43366. doi: 10.1097/MD.0000000000043366 (PMC12282772; doi:10.1097/MD.0000000000043366)
Supplement: Supplementary file 3 [file medi-104-e43366-s003.doc]

**Table S1**

**The GRADE level of evidence**

| **Certainty assessment** | | | | | | | **№ of patients** | | **Effect** | | **Certainty** |
| --- | --- | --- | --- | --- | --- | --- | --- | --- | --- | --- | --- |
| **№ of studies** | **Study design** | **Risk of bias** | **Inconsistency** | **Indirectness** | **Imprecision** | **Other considerations** | **Main outcomes** | **placebo** | **Relative (95% CI)** | **Absolute (95% CI)** |
| Clinical effective rate | | | |  |  |  |  |  |  |  |  |
| 14 | randomised trials | serious^a^ | serious^b^ | not serious | not serious | none | 658/710 (92.7%) | 668/705 (94.8%) | **RR 0.98** (0.95 to 1.00) | **19 fewer per 1,000** (from 47 fewer to 0 fewer) | ⨁⨁◯◯ Low |
| Clinical effective rate - Cooney | | | |  |  |  |  |  |  |  |  |
| 5 | randomised trials | serious^a^ | not serious | not serious | serious^c^ | none | 179/187 (95.7%) | 170/186 (91.4%) | **RR 1.05** (0.99 to 1.11) | **46 more per 1,000** (from 9 fewer to 101 more) | ⨁⨁◯◯ Low |
| Clinical effective rate - Gartland-Werley | | | |  |  |  |  |  |  |  |  |
| 6 | randomised trials | serious^a^ | not serious | not serious | not serious | none | 357/387 (92.2%) | 363/381 (95.3%) | **RR 0.97** (0.93 to 1.00) | **29 fewer per 1,000** (from 67 fewer to 0 fewer) | ⨁⨁⨁◯ Moderate |
| Clinical effective rate - Dinest | | |  |  |  |  |  |  |  |  |  |
| 3 | randomised trials | serious^a^ | not serious | not serious | serious^c^ | none | 122/136 (89.7%) | 135/138 (97.8%) | **RR 0.92** (0.86 to 0.98) | **78 fewer per 1,000** (from 137 fewer to 20 fewer) | ⨁⨁◯◯ Low |
| Radial inclination | |  |  |  |  |  |  |  |  |  |  |
| 7 | randomised trials | serious^a^ | not serious | not serious | not serious | none | 269 | 268 | - | MD **0.1 lower** (0.39 lower to 0.19 higher) | ⨁⨁⨁◯ Moderate |
| Volar tilt | |  |  |  |  |  |  |  |  |  |  |
| 7 | randomised trials | serious^a^ | not serious | not serious | not serious | none | 269 | 268 | - | MD **0.16 lower** (0.34 lower to 0.03 higher) | ⨁⨁⨁◯ Moderate |
| Radial height | |  |  |  |  |  |  |  |  |  |  |
| 3 | randomised trials | serious^a^ | not serious | not serious | serious^c^ | none | 118 | 118 | - | MD **0.09 lower** (0.31 lower to 0.13 higher) | ⨁⨁◯◯ Low |
| Fracture healing time | |  |  |  |  |  |  |  |  |  |  |
| 8 | randomised trials | serious^a^ | serious^b^ | not serious | not serious | none | 409 | 400 | - | MD **1.68 lower** (2.4 lower to 0.96 lower) | ⨁⨁◯◯ Low |
| Complications | |  |  |  |  |  |  |  |  |  |  |
| 10 | randomised trials | serious^a^ | serious^b^ | not serious | serious^c^ | none | 47/430 (10.9%) | 61/426 (14.3%) | **RR 0.82** (0.42 to 1.63) | **26 fewer per 1,000** (from 83 fewer to 90 more) | ⨁◯◯◯ Very low |
| VAS | |  |  |  |  |  |  |  |  |  |  |
| 5 | randomised trials | serious^a^ | serious^b^ | not serious | serious^c^ | none | 174 | 179 | - | MD **0.01 higher** (0.13 lower to 0.16 higher) | ⨁◯◯◯ Very low |

**CI**: confidence interval; **MD**: mean difference; **RR**: risk ratio

**Explanations**

a. No blinding and allocation concealment

b. High heterogeneity

c. Small sample size
